# Effect of Mixed Manure and Inorganic Fertilizer on Phosphorus Adsorption and Desorption Characteristics of Vertisols in Haramaya District, Eastern Ethiopia

**DOI:** 10.1155/2024/4227265

**Published:** 2024-11-13

**Authors:** Dejene Teressa, Kibebew Kibret, Nigussie Dechasa, Lemma Wogi

**Affiliations:** ^1^School of Natural Resources Management and Environmental Sciences, Haramaya University, Dire Dawa, Ethiopia; ^2^School of Plant Sciences, Haramaya University, Dire Dawa, Ethiopia

**Keywords:** availability, combined application, Freundlich adsorption efficiency, quantity, synergistic effect

## Abstract

Applying inorganic phosphorus fertilizer is less effective in increasing crop yields in tropical soils due to precipitation and adsorption reactions. However, research suggests that partial substitution of organic and inorganic fertilizers has shown to improve the efficiency of applied phosphorus fertilizer by reducing its adsorption and enhancing desorption due to their synergistic effects. This study aimed to investigate the impact of treating the soil with mixed manure (MM) rates and blended nitrogen, phosphorus, sulfur, and boron (NPSB) fertilizer on the soil's phosphorus adsorption and desorption characteristics. Results showed increased adsorbed phosphorus in all treatments, with increased added phosphorus (P) concentration from 100 to 500 mg kg^−1^. However, the efficiency of adsorbed P decreased significantly as added P concentration rates increased from 100 to 400 mg·kg^−1^ in all treatments and then decreased as the added P concentration advanced to 500 mg·kg^−1^. Moreover, in all treatments that received combined applications of MM and blended NPSB, both quantity and percentage of desorbed P showed a significant increase. The Freundlich adsorption coefficient and constant were also significantly reduced because of the combined application of MM and blended NPSB, compared to the control and their sole applications. Overall, the soil treated with a combined application of 15 t·ha^−1^ of MM with 100 kg·ha^−1^ of blended NPSB showed the highest reduction in the efficiency of adsorbed P, percentage of desorbed P, Freundlich adsorption capacity, and intensity by 8%, 37.5%, 60%, and 58%, respectively, as compared to the control. These findings indicate that the combined application of MM and blended NPSB can improve the P availability and uptake by maize by reducing its adsorption while increasing desorption characteristics. Finally, this experiment recommends further research on the long-term effects of MM and blended NPSB on P adsorption and desorption characteristics of vertisols.

## 1. Introduction

Phosphorus (P) deficiency has been highlighted as the major impediment to agricultural production in Ethiopia [1, 2]. To overcome this problem, farmers mostly utilize inorganic P fertilizers such as diammonium phosphate, triple super phosphate, blended NPSB, and others. However, due to precipitation and adsorption reactions in the soil, inorganic P fertilization alone has shown to be ineffective for crops. As a consequence, only a small amount of the added P could be utilized by plants [3, 4]. According to Helfenstein et al. [5], less than 20% of applied P in the form of inorganic fertilizer was recovered throughout the crop-growing season. The same authors verified that the remaining amount of the applied P becomes immobile and inaccessible to plant uptake owing to adsorption and precipitation processes in the soil. Similarly, Maluf et al. [6] observed that just 5%–25% of applied inorganic P could be uptake by plants in tropical soils, resulting in low P-fertilizer recovery rates. These findings imply that adsorption and desorption processes in the soil system, which are linked to ion exchange and interactions among the nonlabile, labile, and solution pools, influence P bioavailability [7]. As a consequence, the function of fertilizers in crop production and their value are controlled in part by how the nutrients they contain are available to plants at different stages of growth [8].

The high price of inorganic P fertilizer, along with its low effectiveness (poor crop response), exacerbates the challenges to vertisol's productivity in Ethiopia [9]. Furthermore, organic carbon (OC) content, which is a crucial indication of soil health, was observed to be low [10, 11]. Because of the high concentrations of calcium carbonate, exchangeable calcium (Ca^2+^), and magnesium (Mg^2+^) in the soil, the vertisols of the target research area, including the Lake Haramaya watershed, have a slightly alkaline to alkaline response [12, 13]. At higher pH levels (alkaline reaction), soils having high calcium and magnesium carbonates (CaCO_3_ and MgCO_3_) may adsorb more P and give Ca^2+^ and Mg^2+^ for precipitation processes [7, 14]. This makes P unavailable to plants under an alkaline solution. All of these processes contribute to increased P fixation, making P a critical yield-limiting factor for plant development [15].

Soil amendment using organic materials such as manure is a typical approach for increasing soil OC, P, and other nutrient content to enhance soil fertility and productivity. It has potential to improve crop growth by providing plant nutrients, including micronutrients as well as improve biological activity and physical soil properties such as bulk density, porosity, and hydraulic conductivity and aggregate stability, when compared to conventionally fertilized soils [16, 17]. Furthermore, organic substances have shown to improve P availability and activation coefficient by decreasing the intensity of P adsorption and the maximal phosphate buffering capacity, resulting in some P desorption [18]. Manure has been observed to release organic acids during decomposition, which may improve P availability by lowering the fixation of applied P via competing with PO_4_^−3^ ions for retention sites [19, 20]. The negatively charged organic substance adsorbs or complexes cations Ca^2+^ and Mg^2^ in such alkaline soils, reducing their activity in soil solution and their function in P adsorption and/or precipitation [21, 22]. Furthermore, the dissolution impact of organic acids may enhance the availability of P [23]. Organic material breakdown may result in the emission of CO_2_, which generates carbonic acid and solubilizes some primary minerals containing P [24, 25]. However, organic fertilizer alone is said to be insufficient and sluggish in releasing P to crops [26, 27]. Therefore, partial substitution of an inorganic fertilizer with organic substances such as manure in tropical soils could be very effective in improving the efficiency of applied P by changing its adsorption and desorption processes due to their synergistic effects [16, 28].

While many Ethiopian researchers have examined the P adsorption properties of various soils (e.g., [29–31]), there is insufficient scientific evidence regarding the impact of using mixed manure (MM) in conjunction with the inorganic fertilizer on vertisols' P adsorption and desorption properties. Therefore, we hypothesized that combining MM with blended NPSB fertilizer might increase P availability by altering its adsorption and desorption processes. This research was conducted to explore up-to-date scientific evidence on the influence of MM and blended NPSB on vertisols' P adsorption and desorption properties. The findings of this research might be utilized to enhance the efficiency of P fertilizer via manure incorporation and prevent wastage of important fertilizer sources.

## 2. Materials and Methods

### 2.1. Description of the Experimental Site

**Figure 1:**
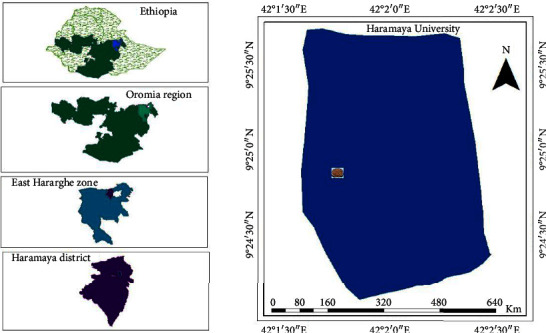
Map of the study area: Haramaya District, Eastern Ethiopia.

The study was conducted on vertisol collected from the crop research site of the main campus of Haramaya University, which is located at a distance of 510 km from Addis Ababa in the east direction. The site is located at 9° 26′ N latitude and 42° 05′ E longitude at an altitude of 2001 m above sea level (Figure 1). The mean annual rainfall of the study area was 500–800 mm from 1995 to 2017 with the peak in August (Ethiopian National Meteorology Agency cited in [32]), and the rainfall distribution pattern is a bimodal type. The short rainy season usually starts in March and ends in May, and the long rainy season (*Ganna or Kiremt*) is between June and September [33].

**Figure 2:**
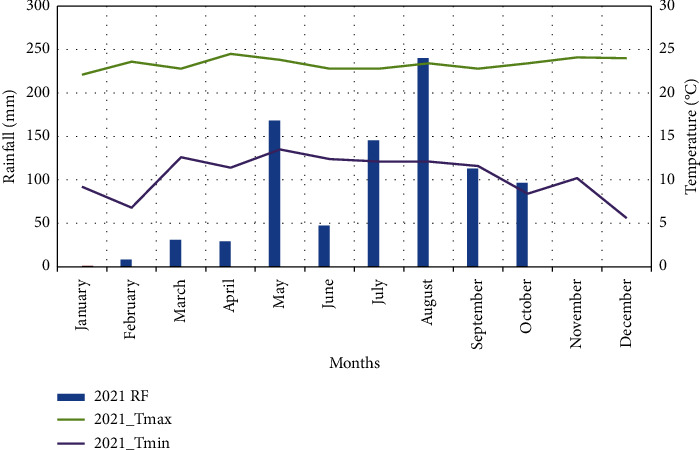
Monthly rainfall (mm) and monthly mean maximum and minimum temperatures (°C) of the experimental site during the 2021 cropping season (source: Jijiga Meteorological Branch Office, Ethiopian National Meteorology Institute).

In 2021, the amount of total annual rainfall was 879.2 mm, with 546 mm during the growing season from sowing to harvest (Figure 2). The maximum and minimum average air temperature ranges during the growing seasons were 22.95°C and 12.05°C, respectively (Figure 2). The historical record of soil management practices at the experimental site, preceding soil sample collection, indicates exclusive use of inorganic fertilizers, specifically urea and DAP. There is no documentation supporting the application of organic fertilizers on the experimental plot. The major soil type of the study area was classified as vertisols [34].

The major food crops grown are cereals (mainly *Sorghum bicolor* and *Zea mays*), pulses (*Phaseolus vulgaris*), and vegetables such as *Allium cepa*, *Solanum tuberosum*, *Daucus carota*, *Brassica oleracea*, *shallot*, and *Capsicum*. Intercropping and alley cropping are also common practices in nearby areas [12].

### 2.2. Experimental Materials and Methods

#### 2.2.1. Experimental Materials

The experimental materials used in this experiment are soil samples collected from plots amended with combined application of MM and blended NPSB fertilizer as presented in Section 2.2.2. The composted MM consisting of 60%, 20%, and 20% of cattle, goat, and poultry manures were used. The proportion of manures with lower calcium content was increased in the mixture since calcium is the primary cause of P adsorption [33].

#### 2.2.2. Treatments and Experimental Design

The treatments consist of soil amended with four rates of MM (0, 5, 10, and 15 t·ha^−1^) and four rates of blended NPSB (0, 50, 100, and 150 kg·ha^−1^) in factorial combinations replicated three times as presented in Table 1. These rates of MM were taken after reviewing different articles as an average.

#### 2.2.3. Soil Sample Collection Analyses

Composite soil samples were collected from five auger points in each plot after maize harvest using the following “zig-zag pattern” and then air-dried, crushed, and passed through a 2-mm-diameter sieve to observe the effects of treatments on soil P adsorption and desorption properties.

#### 2.2.4. Phosphorous Adsorption and Desorption Analysis

Adsorption and desorption studies were conducted using the batch equilibrium method [18, 35, 36]. Plastic bottles of 100 mL capacity were used for each treated soil. To each bottle, 2.50 g of air-dried soil and 25 mL of 0.01 M potassium chloride (KCl) solution containing 0, 10, 20, 30, 40, and 50 mg·L^−1^ P concentration or 0, 100, 200, 300, 400, and 500 mg of P kg soil^−1^ were added to the respective bottle labeled for each P concentration. Two to three drops of phenol were added to inhibit any microbial growth. The mixtures were shaken for 24 h with a speed of 350 rpm at 25 ± 1°C and then equilibrated for 30 min. After equilibration, the suspension was filtered through Whatman filter paper No. 42. One control sample with only P in 0.01 M KCl solution (no soil) was also subjected precisely to the same procedure as the test systems to check the stability of the test substance in the KCl solution and its possible adsorption on the surface of the bottles. The P content in the filtrate was determined calorimetrically using the ascorbic acid-molybdophosphate blue method by spectrophotometer. After the adsorption experiment was completed, the supernatant was removed by adding 25 mL saturated NaCl solution at pH 7.0 to extract the adsorbed P. The solution was then removed, and this process was repeated two times. Then, 25 mL of 0.01M KCl solution at pH 7.0 was added to replace the adsorbed P. The tubes were shaken, centrifuged, and then determined as described above. This P concentration of the solution was defined as the desorbed P [18, 35].

The amount of P adsorbed by the soil was calculated from the differences between the amounts found in the filtrate and the initial amount in the solution using the following equation [37].

(1) $$\begin{array}{c} Q=\left(C_{o}-C_{e}\right)\times \frac{V}{M}, \end{array}$$

where *Q* (mg/kg) is the amount of P adsorbed by the solid phase of soil; *C*_*o*_ and *C*_*e*_ (mg L^−1^) are the initial and equilibrium P concentration, respectively; *V* and *M* are the solution volume and mass of the soil used, respectively.

(2) $$\begin{array}{c} \text{Phosphorus adsorption efficiency}=\frac{C_{o}-C_{e}}{C_{e}}\times 100, \end{array}$$

(3) $$\begin{array}{c} \text{Percentes of }P\text{ desorption}=\frac{\text{amount of desorbed }P}{\text{amount of adsorbed }P}\times 100. \end{array}$$

The P adsorption data were fitted into linearized forms of the Langmuir and Freundlich adsorption equations, separately.

The Langmuir equation can be written in the following linear form by [38]:

(4) $$\begin{array}{c} \frac{1}{Q}=\left(\frac{1}{K_{L}Q_{m}}\right)\frac{1}{C_{e}}+\frac{1}{Q_{m}}, \end{array}$$

where *Q*_*m*_ is the maximum P adsorption (mg kg^−1^); *K*_*L*_ is the binding energy constant, with a maximum quantity consistent with higher soil P adsorption. The *Q*_*m*_ and *K*_*L*_ were obtained by regressing 1/*Q* against 1/*C*_*e*_. *Q*_*m*_ is the reciprocal of the intercept and the *K*_*L*_ is the ratio of intercept to slope [8, 35].

The logarithmic form of the Freundlich isotherm model is as follows:

(5) $$\begin{array}{c} \mathrm{Log}\;Q=\mathrm{Log}\;K_{f}+\frac{1}{n}\mathrm{Log}\;C_{e}, \end{array}$$

where *K*_*f*_ (L mg−^1^) is the Freundlich adsorption coefficient which represents adsorption capacity, whereas *n* is a constant reflecting the adsorption intensity. It is the proportionality constant (mg kg^−1^), *K*_*f*_ = antilog (*Y*-intercept). The 1/*n* is the slope of the curve when log (*Q*) versus log *C*_*e*_ was plotted.

#### 2.2.5. Statistical Analysis

Data obtained were statistically analyzed using an R-statistical software program (version 4.0.5; R Core Team, 2020) by loading appropriate libraries (readr, ggplot2, multcompView, multcomp dplyr, datasets, and agricolae). Three factors analysis of variance (ANOVA) for the quantity and efficiency of adsorbed P and quantity and percentage of desorbed P while two factors ANOVA used for analyzing the Langmuir and Freundlich adsorption isotherms' parameters. The mean comparison was performed using Tukey's HSD test at *p* ≤ 0.05. The regression analysis was also used to compare the fitness of adsorption isotherms.

## 3. Results and Discussion

### 3.1. Physical and Chemical Properties of the Soil and Manures

Selected properties of the experimental soil are presented in Table 2. The textural class of the soil is clay as per the classification of USDA [45]. The bulk density of the soil falls in the range of 1.2–1.4 g cm^−3^ which is classified as some too compact according to Hunt and Gilkes [44]. The soil reaction (pH H_2_O = 7.76) was in the range of slightly alkaline based on Tekalign et al.'s [40] classification. The OC and total N contents of the soil were low and medium, respectively [40]. Available P was also in the range of medium [41]. The CEC of the soil in the study area was very high according to the rating of Landon [42]. The exchangeable Ca and Mg were also very high, with exchangeable K being high [42]. The calcium carbonate content of the soil was also high (12.5%) according to the rating of Bashour and Sayegh [43] which might be responsible for P adsorption.

The results of laboratory analyses of manure from different sources used in the experiment are illustrated in Table 3. The pH value of cattle manure was in the range of neutral while that of poultry and goat manure was alkaline in reactions. The goat manure had a high OC content followed by cattle and poultry, relatively. Poultry manure contained relatively the highest P, nitrogen, Ca, Mg, and K followed by goat manure (Table 3).

### 3.2. The Effects of Mixed Manure and Blended NPSB on the P Adsorption Characteristics of the Vertisols After Maize Harvest

#### 3.2.1. The Amount (Quantity) and Efficiency of Adsorbed P

The results revealed that the interaction effects of MM, NPSB, and added P concentration influenced both the quantity and efficiency of adsorbed P significantly (*p* ≤ 0.001) (Table 4). The quantity of adsorbed P in all soil treated with a combination of MM and NPSB increases with an increased rate of added P concentration from 100 to 500 mg·kg^−1^ (Table 5). However, the efficiency of adsorbed P decreases as the concentration of added P increases from 100 to 400 mg·kg^−1^ in all treatments (Table 6). As added P concentration further increased from 400 to 500 mg·kg^−1^, the efficiency of adsorbed P tends to increase in all treatments except the control (T1), sole 5 t·ha^−1^ of MM (T5), and 50 kg·ha^−1^ of NPSB (T2). This indicates that there is no further P adsorption due to the full saturation of the adsorption sites. The control group (T1) showed the highest amount of P adsorption when 500 mg·kg^−1^ of P was added, while the lowest amount was observed in T15 (15 t·ha^−1^ of MM + 100 kg·ha^−1^ of NPSB) when 100 mg·kg^−1^ of P was added. However, the highest adsorption efficiency was observed in the control group (T1) when 100 mg·kg^−1^ of P was added, while the lowest was observed in T15 (15 t·ha^−1^ of MM + 100 kg·ha^−1^ of blended NPSB) when 400 mg·kg^−1^ of P was added. Overall, adsorbed P's efficiency decreased as the MM and NPSB rates increased in each treatment combination.

Moreover, the combined application of MM and blended NPSB led to a consistent decrease in the overall mean of both the quantity and efficiency of adsorbed P when compared to their respective control treatments (Tables 5 and 6, respectively). Among all the combinations tested, the application of 15 t·ha^−1^ MM with 100 kg·ha^−1^ NPSB (T15) resulted in the highest reduction in the overall means of both quantity and efficiency of adsorbed P, followed by T16 (15 t·ha^−1^ MM + 150 kg·ha^−1^ NPSB). As the rate of MM and blended NPSB increased in each combination, the overall mean values of both quantity and efficiency of adsorbed P progressively decreased.

The observed decrease in P adsorption efficiency could be attributed to the saturation of adsorption sites due to increased P concentration, blended NPSB, and MM, both in combinations and individual applications. All sources of P used in this experiment have resulted in the reduction of P adsorption efficiency. But an integrated application of MM with blended NPSB showed the greatest effect due to their complementary role in increasing P status directly and indirectly through changes in other soil properties such as pH, mineral composition, and OC content.

Consistent with this result, Otieno et al. [46] found that a combination of manure and inorganic fertilizer significantly increases labile P concentrations by reducing P adsorption. Similarly, Azeez and van Averbeke [47] reported a significant reduction in P adsorption efficiency of soil in weathered tropical soil as the result of manure application. Applying manure along with inorganic P fertilizer can increase the amount of negatively charged organic matter in the soil. This organic matter may adsorb complex cations such as calcium (Ca) and magnesium (Mg) in alkaline soils. As a result, their activities in solution (P adsorption and precipitation) could be reduced [21, 22]. In addition, the incorporation of manure into the soil results in the release of organic acids such as humic acid, fulvic acid, and citric acid during the decomposition of organic compounds reported to dissolve calcium phosphate, especially citrate [48]. These organic acids present on the surface of (hydro) oxides can inhibit phosphate adsorption by competing with PO_4_^3−^ ions [49–51]. Nur et al. [52] concurred that manure applied to the soil during decomposition produces humic acid and fulvic acid, which can chelate calcium in the soil. This decrease in P adsorption by Ca subsequently increases the availability of P. Furthermore, the breakdown of organic material releases CO_2_, which generates carbonic acid and solubilizes some primary minerals containing P, highlighting the possibility of reducing P adsorption by using organic compounds [24].

Moreover, Yang et al. [18] reveal that the percentage of adsorbed P decreases as the concentration of added P increases. Similarly, Islam et al. [53] and Bereket et al. [30] confirm that the quantity of P adsorbed by soils increases with increasing levels of added P, while the percentage of added phosphate adsorbed by soils decreases with the increased level of P addition. It has been observed that chemical adsorption is the dominant process at relatively low added P concentrations due to ion exchange and ligand exchange were probably the dominant mechanisms contributing to the high adsorption rate [18, 54]. However, as the concentration of P in the soil solution increases, the process of chemical adsorption slows down rapidly due to the saturation of existing adsorption sites. Consequently, the P present in the soil solution adsorbs to the surface of the soil physiochemical at a relatively slow rate [55, 56].

#### 3.2.2. P Adsorption Isotherms and Parameters

Phosphorus adsorption reactions from various treatments were mathematically described using the Freundlich and Langmuir adsorption isotherms, and the findings are summarized in Table 7. Both Freundlich and Langmuir isotherms were fitted to data obtained from all treatments, with coefficients of determination (*R*^2^) ranging from 0.92 to 0.98 and 0.88 to 0.99, respectively. However, the Freundlich isotherm was the best-fitted model for all treatments, with a greater coefficient of determination (*R*^2^ ≥ 92) than the Langmuir isotherm.

The nutrient adsorption behaviors in soils and their availability to plants have been described by various equations, such as Freundlich and Langmuir isotherms, which are mostly used to understand the relationship between the amounts of P adsorbed per unit soil weight and its concentration in solution [57, 58]. Isotherms are deemed well-fitted if their coefficient of determination (*R*^2^) value is more than 0.9 [38, 59]. As a result, the Freundlich isotherm was discovered to have a higher *R*^2^ value and was thus utilized to characterize the outcomes in this experiment (Table 6). According to Dada et al. [60] and Ayawei et al. [61], the Freundlich isotherm describes the presence of many sites or heterogeneous adsorbent surfaces with diverse adsorption energies. Azeez and van Averbeke [47] also observed similar results for South African soil, where the Freundlich equation had a higher coefficient of determination (*R*^2^) than the Langmuir equation. Similarly, Dubus and Becquer [62] observed comparable results for a variety of Australian soils where P-sorption data were better described by the Freundlich equation.

Freundlich adsorption parameters such as adsorption coefficient (*K*_*f*_) and constant (*n*), signifying adsorption capacity and intensity, respectively, were substantially (*p* ≤ 0.001) influenced by the interaction effects of MM and blended NPSB treatments (Table 8). Except for T5 (5 t·ha^−1^ MM), all treatments' *K*_*f*_ values were significantly lower than the control (T1), with the greatest reduction from T15 (15 t·ha^−1^ MM + 100 kg·ha^−1^ NPSB), which is statistically close to T16 (15 t·ha^−1^ MM + 150 kg·ha^−1^ NPSB) (Table 9). However, in comparison to T4 (full dosage of NPSB), all treatments except T1 (control), T2 (50 kg·ha^−1^ NPSB), T3 (100 kg·ha^−1^ NPSB), T5 (5 t·ha^−1^ MM), T6 (5 t·ha^−1^ MM + 50·kg ha^−1^ NPSB), and T13 (15 t·ha^−1^  MM) had lower *K*_*f*_ values. The leading treatment (T15) showed a 60% and 51% drop in *K*_*f*_ value when compared to the control (T1) and a full dosage of blended NPSB (T4).

Similarly, except T2 (50 kg·ha^−1^ NPSB), T3 (150 kg·ha^−1^ NPSB), and T5 (5 t·ha^−1^ MM), the P adsorption intensity (*n*) of all treatments that received solo or combined application of MM and blended NPSB was considerably lower than the control (T1) (Table 9). The soil treated with a combined application of 15 t·ha^−1^ MM with 100 kg·ha^−1^ of NPSB (T15) had the greatest reduction in *n* value, which is statistically equivalent to T11 (10 t·ha^−1^ MM + 100 kg·ha^−1^ NPSB), T12 (10 t·ha^−1^ MM + 150 kg·ha^−1^ NPSB), and T16 (15 t·ha^−1^ MM + 150 kg·ha^−1^ NPSB). However, compared to T4 (full dose NPSB), soil treated with T9 (10 t·ha^−1^ MM), T10 (10 t·ha^−1^ MM + 50 kg·ha^−1^ NPSB), T11 (10 t·ha^−1^ MM + 100 kg·ha^−1^ NPSB), T12 (10 t·ha^−1^ MM + 150 kg·ha^−1^ NPSB), T15 (15 t·ha^−1^·ha^−1^ MM + 100 kg·ha^−1^ NPSB), and T16 (15 t·ha^−1^ MM + 150 kg·ha^−1^ NPSB) were lowered in their *n* value. The leading treatment (T15) reduced the *n* value of the soil by 58% and 49%, compared to the control (T1) and a full dose of blended NPSB (T4), respectively. These substantial reductions in P adsorption capacity (*K*_*f*_) and intensity (*n*) may be attributed to the synergistic effects of each P source, which increase available P more efficiently than their sole applications.

By adding organic materials from applied manure or indirectly affecting other soil characteristics, including pH, mineral composition, and OC, manure application improves soil P status. Blended NPSB also enhances the amount of easily accessible P in the soil solution. Therefore, by saturating adsorption sites, these elevated P concentrations in the soil solution may lessen the adsorption capacity and intensity. Soil with lower *K*_*f*_ and *n* values is expected to have lower adsorption capacity and intensity than soil with larger *K*_*f*_ and *n* values, indicating that the majority of ions present in the system remain in the solution and are available for transport, chemical processes, and plant uptake. Conversely, higher values of *K*_*f*_ and *n* signify lower mobility and higher ion retention in the soil. Therefore, the higher *K*_*f*_ value suggests greater P adsorption. When the P supply becomes less intense, spontaneous adsorption will occur more quickly [56]. In agreement with this result, Yan et al. [63] found 42.69% and 33.09% lowered *K*_*f*_ mean values by applying poultry manure and inorganic fertilizer compared to the control, respectively. These authors concluded that the buildup and transformation of P in manured soil significantly affected the P-retention capacity, which was supported by changes in the sorption coefficient (*n*) and levels of P saturation. Azeez and van Averbeke [47] also reported lower soil P adsorption capability because of cow, goat, and poultry manure treatments. Similarly, Bahl and Toor [49] also noted a reduction in P-sorption strength in four manure-treated soils. The use of farmyard manure significantly decreased the Freundlich adsorption capacity continuously, according to Sharma et al. [64]. In contrast to these findings, Ahmed et al. [35] reported the highest adsorption capacity from NPK and manure-treated soil compared to sole inorganic fertilizer and control. As a result, the influence of organic sources on P sorption was determined by their P concentration. In this regard, it was suggested that P adsorption increased when the residue had less than 0.22% P and reduced when the residue included more than 0.31% P.

### 3.3. The Effects of Mixed Manure and Blended NPSB Fertilizers on P Desorption Characteristics of Vertisols

#### 3.3.1. Quantity and Percentage of P Desorbed

The interaction effects of MM and blended NPSB demonstrated a substantial (*p* ≤ 0.001) influence on both the quantity and percentage of desorbed P (Table 4). The interaction effects of the treatments demonstrated a substantial (*p* ≤ 0.001) and constant rise in the quantity of desorbed P when the added P concentration rose from 100 to 500 mg·kg^−1^ (Table 10). However, the percentage of desorbed P increased significantly (*p* ≤ 0.001) as the rate of added P concentration increased from 100 to 400 mg·kg^−1^ in all treatments (Table 11). The trend was reversed as added P advanced from 400 to 500 mg·kg^−1^ in all treatments except the control (T1) and sole rates of MM (T5, T9, and T13).

Similarly, the overall mean of the quantity and percentage of desorbed P in all treatments was significantly (*p* ≤ 0.001) enhanced compared to the control. The highest values of these parameters were observed from the combined application of 15 t·ha^−1^ MM and 100 kg·ha^−1^ NPSB (T15). However, as compared to T4 (full dose of blended NPSB), all treatments that received T8 (5 t·ha^−1^ MM + 150 kg·ha^−1^ NPSB), T10 (10 t·ha^−1^ MM + 50 kg ha^−1^ NPSB), T11 (10 t·ha^−1^ MM + 100 kg·ha^−1^ NPSB), T12 (10 t·ha^−1^ MM + 150 kg·ha^−1^ NPSB), T15 (15 t·ha^−1^ MM + 100 kg·ha^−1^ NPSB), and T16 (15 t·ha^−1^ MM + 150 kg ha^−1^ NPSB) resulted in a higher overall mean values of both quantity and percentage of desorbed P in addition to T14 (15 t·ha^−1^ MM + 150 kg·ha^−1^ NPSB) for the quantity of desorbed P. The leading treatment (T15) indicated 20% and 39% larger quantities and percentages of desorbed P, respectively, as compared to the full dose of blended NPSB (Tables 10 and 11, respectively). The augmented quantity and percentage of desorbed P that was observed in this experiment may be attributed to the collaborative influence of MM and blended NPSB. These agents have been found to enhance soil physiochemical properties, thus facilitating the observed outcomes.

However, it was noted that the quantity of desorbed P was still considerably lower than the amount of P that was adsorbed. This indicates that the P adsorption process in the soil under investigation is only partially reversible, most likely due to the high content of calcium and magnesium, which are responsible for P precipitation. However, desorption, which refers to the release of P from the soil's surface, is reported to be a more critical process than adsorption because it makes the immobilized P in the soil available for reuse [56].

In line with this finding, Yang et al. [18] found that an increase in the quantity of desorbed P is directly proportional to the rise in SOM content, as both are negatively charged and hence use the same binding sites in soil colloids. Therefore, modifying the soil P concentration and organic matter content using MM and blended NPSB might improve vertisols' P desorption capability. When manure undergoes decomposition, it releases negatively charged functional groups such as carboxylic acid, hydroxide, and phenol. Organic compounds added to the soil from composted manures have shown to compete with P for adsorption on soil particle surfaces by blocking adsorption sites, potentially increasing the rate of P desorption [65]. According to Arai and Sparks [7], and Havlin et al. [66], organic compounds present in soil can stimulate the availability of P through various mechanisms, including anion substitution of phosphate (H_2_PO_4_) at adsorption sites, coating metal oxides with humus to form a protective cover and dissolution of calcium phosphate. McDowell et al. [67] have further reported that the mineralization of P from poultry manure accelerates the rate of desorption. In addition, P desorption was observed to increase in soils treated with animal manure and sewage sludge, owing to their high total P and organic matter content [68]. Erich et al. [69] have also acknowledged that manure-amended soils exhibited higher plant-available and desorbable P than control soil.

Generally, as more P is added to the soil, its ability to bind or adsorb P decreases. This is because the soil colloids' adsorption sites become saturated. This is known as physical adsorption levels, where P that is physically bound to the soil is quickly released [35]. Thus, a high application rate of P can increase the amount of P released into the soil solution [70].

## 4. Conclusion

The findings of this experiment demonstrated that integrated applications of MM with blended NPSB inorganic fertilizers decreased the P adsorption, whereas increased its desorption characteristics of vertisols. Soil treated with a combination of MM and NPSB had a greater quantity and percentage of desorbed P but lower adsorption efficiency than either treatment with the sole application or the control. Furthermore, when the rate of added P concentration increased, the amount of adsorbed P rose gradually in all treatments, but its adsorption effectiveness was reduced due to adsorption site saturation. In general, we found out that all P sources used in this experiment had reduced P adsorption efficiency. An integrated application of MM with the blended NPSB fertilizer performed better due to their complementary/synergistic effects in increasing P status. The combined application of blended NPSB and MM lowered the Freundlich adsorption coefficient (*K*_*f*_) and constant (*n*), suggesting that the majority of the ions present in the system remain in solution and are accessible for transport, chemical reactions, and plant absorption. In general, the results of this experiment showed that the accumulation and transformation of P in amended soil enhanced P availability, as evidenced by a decrease in the amount and efficiency of adsorbed P, sorption coefficients (*K*_*f*_ and n), and an increase in desorbed P. The combined application of 15 t·ha^−1^ MM and 100 kg·ha^−1^ blended NPSB resulted in the greatest change in the aforementioned parameters. Finally, it is suggested that it is critical to increase P availability in vertisols by using MM alongside blended NPSB fertilizer.

## Figures and Tables

**Table 1:** Treatments and their combinations.

Treatment no	Treatment combinations
T1	Absolute control (0, 0)
T2	0 t·ha^−1^ MM + 50 kg·ha^−1^ NPSB
T3	0 t·ha^−1^ MM + 100 kg·ha^−1^ NPSB
T4	0 t ha^−1^ MM + 150 kg·ha^−1^ NPSB
T5	5 t·ha^−1^ MM + 0 kg·ha^−1^ NPSB
T6	5 t·ha^−1^ MM + 50 kg ha^−1^ NPSB
T7	5 t·ha^−1^ MM+100 kg·ha^−1^ NPSB
T8	5 t·ha^−1^ MM +150 kg·ha^−1^ NPSB
T9	10 t·ha^−1^ MM + 0 kg·ha^−1^ NPSB
T10	10 t·ha^−1^ MM + 50 kg·ha^−1^ NPSB
T11	10 t·ha^−1^ MM +100 kg·ha^−1^ NPSB
T12	10 t·ha^−1^ MM + 150 kg·ha^−1^ NPSB
T13	15 t·ha^−1^ MM + 0 kg·ha^−1^ NPSB
T14	15 t·ha^−1^ MM +50 kg·ha^−1^ NPSB
T15	15 t·ha^−1^ MM + 100 kg·ha^−1^ NPSB
T16	15 t·ha^−1^ MM +150 kg·ha^−1^ NPSB

**Table 2:** Selected physical and chemical properties of the experimental soil in Haramaya district in the eastern highlands of Ethiopia during 2021-22.

Property	Mean ± sd1 (*n* = 3)	Rating/class	References
pH	7.76 ± 0.09	Slightly alkaline	Hazelton and Murphy [39]
Organic carbon (%)	1.18 ± 0.08	Low	Tadesse et al. [40]
Total nitrogen (%)	0.099 ± 0.01	Medium	Tadesse et al. [40]
Available P (mg/kg soil)	16.6 ± 4.18	Medium	Cottenie [41]
CEC (cmol(+) mg/kg soil)	40.0 ± 2.73	High	Landon [42].
Exchangeable cations (cmol(+)/kg soil)			
K	0.64 ± 0.07	High	Landon [42].
Ca	30.2 ± 1.79	Very high	Landon [42].
Mg	6.16 ± 0.41	Very high	Landon [42].
CaCO_3_ (%)	12.5 ± 1.99	High	Bashour and Sayegh [43]
Bulk density (g cm^−3^)	1.24 ± 0.1	Some too compact	Hunt and Gilkes [44]
Particle size distribution (%)			
Sand	35 ± 1.32		
Silt	25 ± 0.50		
Clay	40 ± 1.00		
Textural class	Clay		[45]

*Note: n* is the number of replications.

Abbreviation: sd, standard deviation.

**Table 3:** Selected chemical properties of manures used for the experiment in the Haramaya district of the eastern highlands of Ethiopia during 2021-22.

Parameters	Manures		
	Cattle	Poultry	Goat
pH	6.87	8.15	8.57
Electrical conductivity (dS/m)	0.59	1.07	0.86
Organic carbon (%)	29.9	22.04	31.0
Total P (g/kg manure)	5.58	12.8	7.26
Total N (%)	1.73	3.50	2.27
Mg (g/kg manure)	1.23	1.50	1.36
Ca (g/kg manure)	2.14	6.68	2.19
K (g/kg manure)	1.14	1.97	1.73

**Table 4:** Mean squares for the quantity and efficiency of adsorbed P, and quantity *and percentage of desorbed P as influenced by the combined application of MM* and blended NPSB in Haramaya district in the eastern highlands of Ethiopia during 2021-22.

Source of variation	*df*	Mean square			
		Quantity of adsorbed P	Efficiency of adsorbed P	Quantity of desorbed P	Percentage of desorbed P	
MM	3	762⁣^∗∗∗^	1464⁣^∗∗∗^	495⁣^∗∗∗^	887⁣^∗∗∗^	
NPSB	3	927⁣^∗∗∗^	1629⁣^∗∗∗^	488⁣^∗∗∗^	926⁣^∗∗∗^	
Added P	4	814785⁣^∗∗∗^	10420⁣^∗∗∗^	32839⁣^∗∗∗^	7358⁣^∗∗∗^	
MM∗NPSB	9	383.68⁣^∗∗∗^	113.96⁣^∗∗∗^	20⁣^∗∗∗^	53.31⁣^∗∗∗^	
MM: added P	12	9 8.44⁣^∗∗∗^	100.57⁣^∗∗∗^	1.6	53.33⁣^∗∗∗^	
NPSB: added P	12	17⁣^∗∗∗^	76.61⁣^∗∗∗^	6.4⁣^∗∗∗^	49.31⁣^∗∗∗^	
MM: NPSB: added P	36	4 3.61⁣^∗∗∗^	37.37⁣^∗∗∗^	2.5⁣^∗∗∗^	16.58⁣^∗∗∗^	
Residuals	160	1	0.1	0.01	0.01	

Abbreviations: *D*_f_ = degree of freedom; MM = mixed manure; Ns = not significant.

⁣^∗^, ⁣^∗∗^, and ⁣^∗∗^ = Significant at 5%, 1%, and 0.1% probability levels, respectively.

**Table 5:** The quantity of phosphorus adsorbed by vertisols after maize harvest as influenced by the application of mixed manure and blended NPSB fertilizer in the Haramaya district of the eastern highlands of Ethiopia during 2021-22.

Treatments		Added P (mg kg^−1^ of soil)					Overall
MM t ha^−1^	NPSB kg ha^−1^	100	200	300	400	500	
		Adsorbed P (Q) (mg per kg of soil)					
0	0	99.28L	190.61C	278.49t	348.67i	439.56a	271.32^a^
	50	98.87LM	187.65CDE	276.11tuv	346.25i	437.34a	269.26^b^
	100	98.62LM	186.07DEF	273.32uvwx	338.92lm	432.22cd	265.84^cd^
	150	97.71LM	183.20FGH	272.08wxy	338.02lm	430.19cde	264.24^ef^

5	0	99.03LM	189.34CD	276.57tu	345.44ij	436.13ab	269.30^b^
	50	97.29LM	184.99EFG	272.99vwx	342.71jk	433.39bcd	266.27^c^
	100	96.64LM	182.70FGHI	271.00wxy	333.03opq	429.94de	262.66^gh^
	150	95.59M	181.09HIJ	269.35yz	336.86mn	426.07fg	261.79^h^

10	0	96.39LM	185.45EFG	272.61vwxy	338.86lm	430.92cde	264.85^de^
	50	96.34LM	181.19HIJ	270.04xy	334.47nop	428.56ef	262.12^gh^
	100	91.09N	178.91JK	266.06zA	329.97qr	424.53g	258.11^i^
	150	91.85N	179.32IJK	266.45zA	331.76pqr	424.93g	258.86^i^

15	0	98.20LM	187.06DE	273.87uvw	341.49kl	433.60bc	266.84^c^
	50	96.96LM	182.27GHIJ	271.04wxy	336.20mno	430.46cde	263.39^fg^
	100	86.04O	176.45K	262.17B	325.56s	419.95h	254.03^k^
	150	87.15O	177.45K	262.94AB	328.54rs	422.60gh	255.74^j^

Sig. level		⁣^∗∗∗^					⁣^∗∗∗^

CV (%)		0.38					

Tukey's HSD (0.05)		3.51					

*Note:* Different letters in columns and rows, and superscripts show significant differences at *p* ≤ 0.05.

Abbreviations: CV (%), coefficient of variation; HSD (0.05), honest significance difference at *p* ≤ 0.05; MM, mixed manure.

⁣^∗∗∗^Significant at *p* ≤ 0.001.

**Table 6:** Phosphorus adsorption efficiency of vertisols after maize harvest as influenced by the application of mixed manure and blended NPSB fertilizer in the Haramaya district of the eastern highlands of Ethiopia during 2021-22.

Treatment		Added P (mg kg^−1^ of soil)					Overall
MM t ha^−1^	NPSB kg ha^−1^	100	200	300	400	500	
		Phosphorus adsorption efficiency (%)					
0	0	99.27a	95.30h	92.823jkm	87.17ABCDE	87.92yzA	92.50^a^
	50	98.9ab	93.83ij	92.04mnop	86.56CDEF	87.47ABC	91.76^b^
	100	98.63abc	93.05jkl	91.11pqrs	84.73JKLM	86.45CDEF	90.79^cd^
	150	97.7cde	91.6nopqr	90.69rstu	84.50KLMN	86.04FGHI	90.11^e^

5	0	99ab	94.67hi	92.19lmop	86.36DEFG	87.23ABCDE	91.89^b^
	50	97.27def	92.5klmn	91.00pqrst	85.68FGHIJ	86.67BCDEF	90.62^d^
	100	96.63fg	91.35opqrs	90.32stuv	83.27PWR	85.99FGHI	89.51^fg^
	150	95.60gh	90.55rstu	89.79uvw	84.21LMNO	85.21HIJKLM	89.07^h^

10	0	96.37fg	92.73klm	90.86qrst	84.71JKLM	86.19EFGH	90.17^e^
	50	96.37fg	90.58rstu	90.02tuv	83.63NOP	85.71FGHIJ	89.26^gh^
	100	91.10pqrst	89.45vwx	88.68xyz	82.50QR	84.91JKLM	87.32^i^
	150	91.83mnopq	89.67uvwx	88.82wxy	82.94PQR	84.99IJKLM	87.65^i^

15	0	98.17bcd	93.53jk	91.29opqrs	85.38GHIJK	86.72BCDEF	91.02^c^
	50	96.97eg	91.13opqrs	90.35stuv	84.05MNO	86.09FGH	89.72^f^
	100	88.22yzA	86.03FGHI	87.39ABCD	81.39S	83.99MNOP	85.40^k^
	150	88.73wxy	87.17ABCDE	87.66Zab	82.14RS	84.53KLMN	86.05^j^

Level of sig.		⁣^∗∗∗^					⁣^∗∗∗^

CV (%)		0.35					

Tukey's HSD (0.05)		1.11					

*Note:* Different letters in columns and rows, and superscripts show significant differences at *p* ≤ 0.05.

Abbreviations: CV, coefficient of variation; HSD (0.05), honest significance difference at *p* ≤ 0.05; MM: mixed manure.

⁣^∗∗∗^Significant at *p* ≤ 0.001.

**Table 7:** Regression equations and coefficient of determination (*R*^2^) indicate the fitness of phosphorus adsorption data to Langmuir and Freundlich isotherms as influenced by the application of mixed manure and blended NPSB fertilizer in Haramaya district of eastern highlands of Ethiopia during 2021-22.

Treatments		Langmuir isotherms		Freundlich isotherm	
MM t ha^−1^	NPSB kg ha^−1^	1/*Q* = 1/(*Q*_*m*_*K*_1_)∗1/*C*_*e*_ + 1/(*Q*_*m*_)	*R* ^2^	Log *Q* = log *K*_*f*_ + 1/*n* log *C*_*e*_	*R* ^2^
0	0	1/*Q* = 0.0005*C*_*e*_ + 0.0032	0.88⁣^∗∗^	*Q* = 0.3165*C*_*e*_ + 2.3409	0.96⁣^∗∗∗^
	50	1/*Q* = 0.0007*C*_*e*_ + 0.0032	0.88⁣^∗^	*Q* = 0.3425*C*_*e*_ + 2.3100	0.95⁣^∗∗∗^
	100	1/*Q* = 0.0008*C*_*e*_ + 0.0032	0.88⁣^∗^	*Q* = 0.3460*C*_*e*_ + 2.2880	0.95⁣^∗∗∗^
	150	1/*Q* = 0.0012*C*_*e*_ + 0.0032	0.88⁣^∗^	*Q* = 0.3831*C*_*e*_ + 2.2564	0.95⁣^∗∗∗^
5	0	1/*Q* = 0.0005*C*_*e*_ + 0.0027	0.89⁣^∗∗^	*Q* = 0.3322*C*_*e*_ + 2.3188	0.97⁣^∗∗∗^
	50	1/*Q* = 0.0017*C*_*e*_ + 0.0029	0.91⁣^∗^	*Q* = 0.4255*C*_*e*_ + 2.2446	0.97⁣^∗∗∗^
	100	1/*Q* = 0.0020*C*_*e*_ + 0.0029	0.91⁣^∗^	*Q* = 0.4292*C*_*e*_ + 2.2179	0.96⁣^∗∗∗^
	150	1/*Q* = 0.0029*C*_*e*_ + 0.0027	0.93⁣^∗^	*Q* = 0.4808*C*_*e*_ + 2.1767	0.97⁣^∗∗∗^

10	0	1/*Q* = 0.0031*C*_*e*_ + 0.0032	0.95⁣^∗∗^	*Q* = 0.4673*C*_*e*_ + 2.2129	0.98⁣^∗∗∗^
	50	1/*Q* = 0.0023*C*_*e*_ + 0.0029	0.91⁣^∗^	*Q* = 0.4464*C*_*e*_ + 2.1983	0.96⁣^∗∗∗^
	100	1/*Q* = 0.0071*C*_*e*_ + 0.0017	0.99⁣^∗∗^	*Q* = 0.6289*C*_*e*_ + 2.0546	0.96⁣^∗∗∗^
	150	1/*Q* = 0.0062*C*_*e*_ + 0.0019	0.99⁣^∗∗^	*Q* = 0.6098*C*_*e*_ + 2.0742	0.97⁣^∗∗∗^

15	0	1/*Q* = 0.0011*C*_*e*_ + 0.0031	0.90⁣^∗∗^	*Q* = 0.3788*C*_*e*_ + 2.2766	0.97⁣^∗∗∗^
	50	1/*Q* = 0.0017*C*_*e*_ + 0.0030	0.90⁣^∗^	*Q* = 0.4132*C*_*e*_ + 2.2310	0.95⁣^∗∗∗^
	100	1/*Q* = 0.0119*C*_*e*_ + 0.0007	0.96⁣^∗∗^	*Q* = 0.7463*C*_*e*_ + 1.9455	0.92⁣^∗∗∗^
	150	1/*Q* = 0.0109*C*_*e*_ + 0.0009	0.97⁣^∗∗^	*Q* = 0.7299*C*_*e*_ + 1.9704	0.94⁣^∗∗∗^

*Note:C*_*e*_: equilibrium concentration; *K*_1_: Langmuir adsorption constant; *Q*: quantity/amount of adsorbed phosphorus; *Q*_*m*_: maximum phosphorus adsorption; *K*_*f*_: Freundlich adsorption coefficient; *n*: Freundlich adsorption constant.

Abbreviation: MM, mixed manure.

⁣^∗∗∗^Significant at *p* ≤ 0.001.

⁣^∗∗^Significant at *p* ≤ 0.01.

⁣^∗^Significant at *p* ≤ 0.05.

**Table 8:** Mean squares for Langmuir and Freundlich phosphorus adsorption parameters as influenced by combined application of MM and blended NPSB in Haramaya district in the eastern highlands of Ethiopia during 2021-22.

Source of variation	*df*	Mean square				
		*Q*_*m*_	*K*_*L*_	MBC	*K*_*F*_	*n*
MM	3	597851⁣^∗∗∗^	34.17⁣^∗∗∗^	2945666⁣^∗∗∗^	11396⁣^∗∗∗^	2.64⁣^∗∗∗^
NPSB	3	354023⁣^∗∗∗^	20.79⁣^∗∗∗^	1889078⁣^∗∗∗^	9918⁣^∗∗∗^	1.74⁣^∗∗∗^
MM∗NPSB	9	203958⁣^∗∗^	1.97⁣^∗∗∗^	169574⁣^∗∗∗^	934⁣^∗∗∗^	0.21⁣^∗∗∗^
Residuals	32	2661	0.4	32400	14	0.01

Abbreviations: *D*_f_ = degree of freedom; MM = mixed manure; Ns = not significant.

⁣^∗^, ⁣^∗∗^, and ⁣^∗∗^= Significant at 5%, 1%, and 0.1% probability levels, respectively.

**Table 9:** Langmuir and Freundlich P adsorption parameters as influenced by the application of mixed manure and blended NPSB fertilizer in Haramaya district of eastern highlands of Ethiopia during 2021-2022.

Treatments		Langmuir isotherms				Freundlich isotherms		
MM t ha^−1^	NPSB kg ha^−1^	*Q*_*m*_ (mg kg^−1^)	*K*_*l*_ (L mg^−1^)	MBC (L kg^−1^)	*R* ^2^	*K*_*f*_ (mg kg^−1^)	*n* (mg L^−1^)	*R* ^2^
0	0	308.72d	6.80a	2093.32a	0.88⁣^∗∗^	219.23a	3.16a	0.96⁣^∗∗∗^
	50	310.26d	4.59bc	1422.45bc	0.88⁣^∗^	204.18bc	2.92ab	0.95⁣^∗∗∗^
	100	308.09d	4.02bcd	1234.30bcd	0.88⁣^∗^	194.08cd	2.89ab	0.95⁣^∗∗∗^
	150	315.86d	2.59de	816.06def	0.88⁣^∗^	180.45ef	2.61c	0.95⁣^∗∗∗^

5	0	376.89d	5.19ab	1608.45ab	0.89⁣^∗∗^	208.34ab	3.01a	0.97⁣^∗∗∗^
	50	341.43d	1.75ef	594.57efg	0.91⁣^∗^	175.65fg	2.35cde	0.97⁣^∗∗∗^
	100	339.96d	1.48ef	501.09efg	0.91⁣^∗^	165.14ghi	2.33cde	0.96⁣^∗∗∗^
	150	368.45d	0.94ef	344.83fg	0.93⁣^∗^	150.22j	2.08e	0.97⁣^∗∗∗^

10	0	311.72d	1.05ef	395.84efg	0.95⁣^∗∗^	163.27hi	2.14de	0.98⁣^∗∗∗^
	50	345.78d	1.25ef	431.63efg	0.91⁣^∗^	157.88ij	2.24de	0.96⁣^∗∗∗^
	100	589.29c	0.24f	139.44g	0.99⁣^∗∗^	113.40k	1.59f	0.96⁣^∗∗∗^
	150	535.56c	0.30f	157.58g	0.99⁣^∗∗^	118.63k	1.64f	0.97⁣^∗∗∗^

15	0	323.84d	2.82cde	907.32cde	0.90⁣^∗∗^	189.06de	2.64bc	0.97⁣^∗∗∗^
	50	328.61d	1.81ef	592.75efg	0.90⁣^∗^	170.20fgh	2.42cd	0.95⁣^∗∗∗^
	100	1396.41a	0.06f	81.29g	0.96^ns^	88.21L	1.34f	0.92⁣^∗∗∗^
	150	1142.43b	0.08f	90.35g	0.97^ns^	93.42L	1.37f	0.94⁣^∗∗∗^

Sig. level.	⁣^∗∗∗^	⁣^∗∗∗^	⁣^∗∗∗^		⁣^∗∗∗^	⁣^∗∗∗^		

CV (%)	11	29	25		4.59	2.31		

Tukey's HSD (0.05)	156	1.9	545		0.32	11.33		

*Note:* Different capital letters in a column and different small letters in a column show significant differences at *p* ≤ 0.05; honest significance difference at *p* ≤ 0.05; *K*_*l*_: Langmuir adsorption constant; *Q*: quantity/amount of adsorbed phosphorus; *Q*_*m*_: maximum phosphorus adsorption; *K*_*f*_: Freundlich adsorption coefficient; *n*: Freundlich adsorption constant.

Abbreviations: CV, coefficient of variation; MBC, maximum buffering capacity; MM, mixed manure.

⁣^∗∗∗^Significant at *p* ≤ 0.001.

**Table 10:** The quantity of phosphorus desorbed from vertisols application of mixed manure and blended NPSB fertilizer in Haramaya district of eastern highlands of Ethiopia during 2021-22.

Treatments		Added P (mg kg^−1^ of soil)					
MM t ha^−1^	NPSB kg ha^−1^	100	200	300	400	500	Overall
		Quantity of desorbed P (D) in mg kg^−1^ of soils					
0	0	0.47M	4.69HI	17.36y	26.57pq	30.86klm	15.99^L^
	50	0.82LM	6.25EFGH	18.67xy	27.92op	32.84hij	17.30^k^
	100	0.59M	7.43DEF	19.91wx	29.13mno	33.26fghi	18.07^ij^
	150	1.17KLM	9.59ABC	20.77uvw	30.75klm	34.06efghi	19.27^fg^

5	0	0.85LM	5.72FGHI	19.14wxy	28.05nop	33.28fghi	17.41^jk^
	50	1.42KLM	7.82CDE	20.61uvw	29.95lm	34.10efghi	18.78^gh^
	100	1.88KLM	9.84AB	20.88uvw	30.68klm	34.89defg	19.63^ef^
	150	2.79JK	10.48Zab	23.14rst	31.10jkl	34.45efgh	20.39^d^

10	0	1.48KLM	7.21DEFG	20.67uvw	29.81mn	34.82defg	18.80^fg^
	50	2.50JKL	9.31ABC	22.21stu	31.18jkl	35.55bcde	20.15^de^
	100	4.86HI	11.03zA	23.90rs	32.49ijk	37.30ab	21.91^bc^
	150	4.31IJ	11.10zA	24.15r	32.38ijk	36.39bcd	21.67^c^

15	0	1.22KLM	7.36DEFG	20.19vwx	29.35mno	34.10efghi	18.44^hi^
	50	2.25KLM	8.76BCD	21.94tuv	30.87klm	35.10cdef	19.78^def^
	100	6.25EFGH	12.06z	24.76qr	33.94efghi	39.11a	23.22^a^
	150	5.53GHI	11.72z	24.44r	33.23ghi	36.81bc	22.35^b^

Level of sig		⁣^∗∗∗^					⁣^∗∗∗^

CV (%)		0.05					

Tukey's HSD (0.05)		0.04					

*Note:* Different letters in columns and rows, and superscripts show significant differences at *p* ≤ 0.05.

Abbreviations: CV (%), coefficient of variation; HSD (0.05), honest significance difference at *p* ≤ 0.05; MM: mixed manure.

⁣^∗∗∗^significant at *p* ≤ 0.001.

⁣^∗∗^significant at *p* ≤ 0.01.

⁣^∗^significant at *p* ≤ 0.05.

**Table 11:** The quantity of phosphorus desorbed from vertisols as influenced by the application of mixed manure and blended NPSB fertilizer in the Haramaya district of eastern highlands of Ethiopia during 2021-22.

Treatments		Added P (mg kg^−1^ of soil)					
MM t ha^−1^	NPSB kg ha^−1^	100	200	300	400	500	Overall
		Percentage of desorbed P					
0	0	0.48H	2.46CD	6.23tu	7.62jklmno	7.02nopqrs	4.76^k^
	50	0.83FGH	3.33AB	6.76qrst	8.06hijkl	7.51klmnopq	5.30^j^
	100	0.60GH	3.99yzA	7.28mnopqr	8.60fghi	7.69jklmn	5.63^i^
	150	1.19EFGH	5.23vw	7.63jklmno	9.10cdef	7.92ijklm	6.21^fg^

5	0	0.86FGH	3.02BC	6.92opqrst	8.12hijkl	7.63jklmno	5.31^j^
	50	1.46EF	4.23xyz	7.55jklmnop	8.74efgh	7.87ijklm	5.97^gh^
	100	1.96DE	5.38vw	7.70jiklmn	9.21cdef	8.12hijkl	6.47^ef^
	150	2.91BC	5.79uv	8.59fghi	9.23cdef	8.09hijkl	6.92^d^

10	0	1.54EF	3.89 zA	7.58jklmnop	8.80efgh	8.08hijkl	5.98^gh^
	50	2.59BCD	5.14vw	8.23ghijk	9.32cdef	8.29ghij	6.71^de^
	100	5.33vw	6.17tu	8.98efg	9.85ab	8.78efgh	7.82^c^
	150	4.69wxy	6.19tu	9.06def	9.76abc	8.56fghi	7.65^c^

15	0	1.25EFG	3.93yzA	7.38lmnopq	8.59fghi	7.86ijklm	5.80^hi^
	50	2.33CD	4.81wx	8.09hijkl	9.18cdef	8.16hijk	6.51^e^
	100	6.84pqrst	7.27mnopqr	9.44bcde	10.43a	9.31cdef	8.66^a^
	150	6.34stu	6.60rst	9.29cdef	10.11ab	8.72efgh	8.21^b^

Sig. level	⁣^∗∗∗^						⁣^∗∗∗^

CV (%)	1.5						

Tukey's HSD (0.05)	0.4						

*Note:* Different letters in columns and rows, and superscripts show significant differences at *p* ≤ 0.05.

Abbreviations: CV, coefficient of variation; HSD (0.05), honest significance difference at *p* ≤ 0.01; MM, mixed manure; Sig., significant.

⁣^∗∗∗^significant at *p* ≤ 0.001.

## Data Availability

The data that support the findings of this study are available from the corresponding author upon reasonable request.

## References

[1] Adimassu Z., Mekonnen K., Yirga C., Kessler A. (2014). Effect of Soil Bunds on Runoff, Soil and Nutrient Losses, and Crop Yield in the Central Highlands of ethiopia. *Land Degradation & Development*.

[2] Erkossa T., Williams T. O., Laekemariam F. (2018). Integrated Soil, Water and Agronomic Management Effects on Crop Productivity and Selected Soil Properties in Western Ethiopia. *International Soil and Water Conservation Research*.

[3] Gérard F. (2016). Clay Minerals, Iron/Aluminum Oxides, and Their Contribution to Phosphate Sorption in Soils—A Myth Revisited. *Geoderma*.

[4] Zhang J. B., Yang J. S., Yao R. J., Yu S. P., Li F. R., Hou X. J. (2014). The Effects of Farmyard Manure and Mulch on Soil Physical Properties in a Reclaimed Coastal Tidal Flat Salt-Affected Soil. *Journal of Integrative Agriculture*.

[5] Helfenstein J., Tamburini F., von Sperber C. (2018). Combining Spectroscopic and Isotopic Techniques Gives a Dynamic View of Phosphorus Cycling in Soil. *Nature Communications*.

[6] Maluf H. J. G. M., Silva C. A., Curi N., Norton L. D., Rosa S. D. (2018). Adsorption and Availability of Phosphorus in Response to Humic Acid Rates in Soils Limed With CACO3 or MGCO3. *Ciencia E Agrotecnologia*.

[7] Arai Y., Sparks D. L. (2007). Phosphate Reaction Dynamics in Soils and Soil Components: A Multiscale Approach. *Advances in Agronomy*.

[8] Palanivell P., Ahmed O. H., Latifah O., Majid N. M. A. (2020). Adsorption and Desorption of Nitrogen, Phosphorus, Potassium, and Soil Buffering Capacity Following Application of Chicken Litter Biochar to an Acid Soil. *Applied Sciences*.

[9] Misskire Y., Mamo T., Taddesse A. M., Yermiyahu U. (2019). Potassium Adsorption and Release Characteristics on Vertisols of North Western Ethiopian Highlands. *Communications in Soil Science and Plant Analysis*.

[10] Hailu H., Mamo T., Keskinen R., Karltun E., Gebrekidan H., Bekele T. (2015). Soil Fertility Status and Wheat Nutrient Content in Vertisol Cropping Systems of Central Highlands of Ethiopia. *Agriculture & Food Security*.

[11] Mamo T., Richter C., Heiligtag B. (2002). Phosphorus Availability Studies on Ten Ethiopian Vertisols. *Journal of Agriculture and Rural Development in the Tropics and Subtropics*.

[12] Kibebew K. (2014). Characterization of Agricultural Soils in Cascape Intervention Woredas in Eastern Region.

[13] Workineh M., Dechassa N., Sharma J. J. (2019). Influence of Integrated Fertilizer Application on Growth and Tuber Yield of Potato (*Solanum tuberosum* L.) in Haramaya District, Eastern Ethiopia Haramaya University, College of Agriculture. *World Journal of Agricultural Sciences*.

[14] Devau N., Le Cadre E., Hinsinger P., Gérard F. (2010). A Mechanistic Model for Understanding Root-Induced Chemical Changes Controlling Phosphorus Availability. *Annals of Botany*.

[15] Jalali M. (2007). Phosphorus Status and Sorption Characteristics of Some Calcareous Soils of Hamadan, Western Iran. *Environmental Geology*.

[16] Dejene K., Dereje, Asefa D. G. (2010). Synergistic Effect of A Combined Application of Organic and Inorganic Fertilizers on Yield and Yield Components of Tef (Eragrostis Tef (Zucc.) Trotter) under Terminal Drought at Adiha, Northern Ethiopia. *Journal of the Drylands*.

[17] Kugbe J. X. (2019). Increase in the Use of Organic Fertilizers as Complements to Inorganic Fertilizers in Maintenance of Soil Fertility and Environmental Sustainability. *World Journal of Agriculture and Soil Science*.

[18] Yang X., Chen X., Yang X. (2019). Effect of Organic Matter on Phosphorus Adsorption and Desorption in a Black Soil From Northeast China. *Soil and Tillage Research*.

[19] Park M., Singvilay O., Shin W., Kim E., Chung J., Sa T. (2004). Effects of Long-Term Compost and Fertilizer Application on Soil Phosphorus Status under Paddy Cropping System. *Communications in Soil Science and Plant Analysis*.

[20] Xie R. J., MacKenzie A. F., O’Halloran I. P. (1991). Lignosulfonate Effects on Phosphate Reactions in a Clay Soil: Causal Modeling. *Soil Science Society of America Journal*.

[21] Haynes R. J., Mokolobate M. S. (2001). *Amelioration of Al Toxicity and P Deficiency in Acid Soils by Additions of Organic Residues: A Critical Review of the Phenomenon and the Mechanisms Involved*.

[22] Nyle C., Brady R. R. W. (2017). *The Nature and Properties of Soils*.

[23] Meena M. D., Biswas D. R. (2014). Phosphorus and Potassium Transformations in Soil Amended With Enriched Compost and Chemical Fertilizers in a Wheat-Soybean Cropping System. *Communications in Soil Science and Plant Analysis*.

[24] Johan P. D., Ahmed O. H., Omar L., Hasbullah N. A. (2021). Charcoal and Sago Bark Ash on Ph Buffering Capacity and Phosphorus Leaching. *Agronomy*.

[25] Oburger E., Jones D. L., Wenzel W. W. (2011). Phosphorus Saturation and pH Differentially Regulate the Efficiency of Organic Acid Anion-Mediated P Solubilization Mechanisms in Soil. *Plant and Soil*.

[26] Pinitpaitoon S., Suwanarit A., Bell R. W. (2011). A Framework for Determining the Efficient Combination of Organic Materials and Mineral Fertilizer Applied in Maize Cropping. *Field Crops Research*.

[27] Vanlauwe B., Giller K. E. (2006). Popular Myths Around Soil Fertility Management in Sub-Saharan Africa. *Agriculture, Ecosystems & Environment*.

[28] Alemu M. M. (2015). Effect of Tree Shade on Coffee Crop Production. *Journal of Sustainable Development*.

[29] Abdenna D., Muktar M., Lemma W. (2020). Phosphorus Sorption Characteristics of Lime Amended Ultisols and Alfisols in Humid Tropical Western Ethiopia. *Environmental and Soil Science*.

[30] Ayenew B., Tadesse A. M., Kibret K., Melese A. (2018). Phosphorous Status and Adsorption Characteristics of Acid Soils From Cheha and Dinsho Districts, Southern Highlands of Ethiopia. *Environmental Systems Research*.

[31] Zinabu Wolde W. H. D. S. (2015). Phosphorus Sorption Characteristics and External Phosphorus Requirement of Bulle and Wonago Woreda, Southern Ethiopia. *Advances in Crop Science and Technology*.

[32] Mosisa W., Dechassa N., Kibret K., Zeleke H., Ekeko Bz. (2022). Effects of Timing and Nitrogen Fertilizer Application Rates on Maize Yield Components and Yield in Eastern Ethiopia. *Agrosystems, Geosciences and Environment*.

[33] Teressa D., Kibret K., Dechasa N., Wogi L. (2024). Heliyon Soil Properties and Nutrient Uptake of Maize (*Zea mays*) as Influenced by Mixed Manure and Blended Inorganic Fertilizer in Haramaya District, Eastern Ethiopia. *Heliyon*.

[34] Gezahegn A. M., Tesfaye K., Sharma J. J., Belel M. D. (2016). Determination of Optimum Plant Density for Faba Bean (Vicia faba L.) on Vertisols at Haramaya, Eastern Ethiopia. *Cogent Food & Agriculture*.

[35] Ahmed W., Jing H., Kailou L. (2021). Impacts of Long-Term Inorganic and Organic Fertilization on Phosphorus Adsorption and Desorption Characteristics in Red Paddies in Southern China. *PLoS One*.

[36] Yan X., Wang D., Zhang H., Zhang G., Wei Z. (2013). Organic Amendments Affect Phosphorus Sorption Characteristics in a Paddy Soil. *Agriculture, Ecosystems & Environment*.

[37] Jamiu O. A., Ibijolaa T. O., Michael T., Adetunjia M., Oyekanmi A. A. & A. A. (2014). Chemical Characterization and Stability of Poultry Manure Tea and Its Influence on Phosphorus Sorption Indices of Tropical Soils. *Communications in Soil Science and Plant Analysis*.

[38] Alfaro-Cuevas-Villanueva R., Hidalgo-Vázquez A. R., Cortés Penagos C. D. J., Cortés-Martínez R. (2014). Thermodynamic, Kinetic, and Equilibrium Parameters for the Removal of Lead and Cadmium From Aqueous Solutions With Calcium Alginate Beads. *The Scientific World Journal*.

[39] Hazelton P., Murphy B. (2007). Interpreting Soil Test Results: What Do All the Numbers Mean?. *European Journal of Soil Science*.

[40] Tadesse T., Haque I., Aduayi E. A. (1991). Soil, Plant, Water, Fertilizer, Animal. *Manure and Compost Analysis Manual*.

[41] Cottenie A. (1980). Soil and Plant Testing and Analysis as a Basis of Fertilizer Recommendations. *FAO Soils Bulletin No.*.

[42] Landon J. R. (1991). *Booker Tropical Soil Manual: A Handbook for Soil Survey and Agricultural Land Evaluation in the Tropics and Subtropics. Longman Scientific & Technical*.

[43] Bashour I. I., Sayegh A. H. (2007). *Methods of Analysis for Soils of Arid and Semi-arid Regions*.

[44] Hunt N., Gilkes B. (1992). Soil Structure and Drainage. *Farm Monitoring Handbook*.

[45] Soil Survey Staff U. (1999). Soil Taxonomy: A Basic System of Soil Classification for Making and Interpreting Soil Surveys. *Agriculture Handbook, Number 436*.

[46] Otieno E. O., Lenga F. K., Mburu D. M., Kiboi M. N., Andreas Fliessbach F. K. N. (2023). Influence of Soil Fertility Management Technologies on Phosphorus Fractions, Sorption Characteristics, and Use Efficiency in Humic Nitisols of Upper Eastern Kenya. *Heliyon*.

[47] Azeez J. O., van Averbeke W. (2011). Effect of Manure Types and Period of Incubation on Phosphorus-Sorption Indices of a Weathered Tropical Soil. *Communications in Soil Science and Plant Analysis*.

[48] Shen J., Yuan L., Zhang J. (2011). Phosphorus Dynamics: From Soil to Plant. *Plant Physiology*.

[49] Bahl G. S., Toor G. S. (2002). Influence of Poultry Manure on Phosphorus Availability and the Standard Phosphate Requirement of Crop Estimated From Quantity-Intensity Relationships in Different Soils. *Bioresource Technology*.

[50] Borggaard O. K., Raben-Lange B., Gimsing A. L., Strobel B. W. (2005). Influence of Humic Substances on Phosphate Adsorption by Aluminium and Iron Oxides. *Geoderma*.

[51] Gorgin N., Fekri M., Sadegh L. (2011). Impact of Organic-Matter Application on Phosphorus-Desorption Kinetics in Two Agricultural Soils in Southeastern Iran. *Communications in Soil Science and Plant Analysis*.

[52] Nur M. S. M., Arsa I. G. B. A., Malaipada Y. (2019). The Effect of Cattle Manure and Mineral Fertilizers on Soil Chemical Properties and Tuber Yield of Purple-Fleshed Sweet Potato in the Dryland Region of East Nusa Tenggara, Indonesia. *Tropical Drylands*.

[53] Islam A. U. A., Naher A. U., Hossein A. T. M., Miitra S. B. K., Saleque M. A. (2004). Effect of Organic and Inorganic Amendment on Phosphorus Sorption Characteristic of Lowland Soil. *Bangladesh Journal of Agricultural Research*.

[54] Jiang Y., Yan Q., Liu T., Xu Y. (2023). Phosphorus Adsorption Characteristics and Release Risk in Saline Soils: A Case Study of Songnen Plain, China. *Frontiers in Plant Science*.

[55] Lai D. Y. F., Lam K. C. (2009). Phosphorus Sorption by Sediments in a Subtropical Constructed Wetland Receiving Stormwater Runoff. *Ecological Engineering*.

[56] Wang L., Liang T. (2014). Effects of Exogenous Rare Earth Elements on Phosphorus Adsorption and Desorption in Different Types of Soils. *Chemosphere*.

[57] Kassa M., Haile W., Kebede F. (2019). Evaluation of Adsorption Isotherm Models for Potassium Adsorption under Different Soil Types in Wolaita of Southern Ethiopia. *Communications in Soil Science and Plant Analysis*.

[58] Lulu M., Lemma B., Melese A. (2022). Phosphorous Sorption Characteristics of Soils in Smallholding Land Use in Southern Ethiopia. *Applied and Environmental Soil Science*.

[59] Okeola F. O., Odebunmi E. O. (2010). Freundlich and Langmuir Isotherms Parameters for Adsorption of Methylene Blue by Activated Carbon Derived from Agrowastes. *Advances in Natural and Applied Sciences*.

[60] Dada A. O., Ojediran J. O., Olalekan A. P. (2013). Sorption of Pb2+ From Aqueous Solution unto Modified Rice Husk: Isotherms Studies. *Advances in Physical Chemistry*.

[61] Ayawei N., Ebelegi A. N., Wankasi D. (2017). Modelling and Interpretation of Adsorption Isotherms. *Journal of Chemistry*.

[62] Dubus I. G., Becquer B. (2001). Phosphorus Sorption and Desorption in Oxide-Rich Ferralsols of New Caledonia. *Australian Journal of Soil Research*.

[63] Yan Z., Chen S., Dari B., Sihi D., Chen Q. (2018). Phosphorus Transformation Response to Soil Properties Changes Induced by Manure Application in a Calcareous Soil. *Geoderma*.

[64] Sharma K. R., Srivastava P. C., Srivastava P., Singh V. P. (2006). Effect of Farmyard Manure Application on Boron Adsorption-Desorption Characteristics of Some Soils. *Chemosphere*.

[65] Shariatmadari H., Shirvani M., Jafari A. (2006). Phosphorus Release Kinetics and Availability in Calcareous Soils of Selected Arid and Semiarid Toposequences. *Geoderma*.

[66] Havlin J. L., Beaton J. D., Tisdale S. L., Nelson W. R., Nelson W. L. (2017). Soil Fertility and Fertilizers: An Introduction to Nutrient Management Title Soil Fertility and Fertilizers.

[67] McDowell R. W., Mahieu N., Brookes P. C., Poulton P. R. (2003). Mechanisms of Phosphorus Solubilisation in a Limed Soil as a Function of pH. *Chemosphere*.

[68] Hosseinpur A., Pashamokhtari H. (2008). Impact of Treated Sewage Sludge Application on Phosphorus Release Kinetics in Some Calcareous Soils. *Environmental Geology*.

[69] Erich M. S., Fitzgerald C. B., Porter G. A. (2002). The Effect of Organic Amendments on Phosphorus Chemistry in a Potato Cropping System. *Agriculture, Ecosystems & Environment*.

[70] Johan P. D., Ahmed O. H., Omar L., Hasbullah N. A. (2021). Phosphorus Transformation in Soils Following Co-Application of Charcoal and Wood Ash. *Agronomy*.

